# Modeling miRNA-mRNA interactions: fitting chemical kinetics equations to microarray data

**DOI:** 10.1186/1752-0509-8-19

**Published:** 2014-02-18

**Authors:** Zijun Luo, Robert Azencott, Yi Zhao

**Affiliations:** 1 Department of Mathematics, University of Houston, 4800 Calhoun, Houston, TX, USA; 2 School of Natural Sciences and Humanities, Harbin Institute of Technology, Shenzhen Graduate School, Shenzhen, Guangdong, China

**Keywords:** miRNA, Chemical kinetics modeling, Minimal net clustering

## Abstract

**Background:**

The miRNAs are small non-coding RNAs of roughly 22 nucleotides in length, which can bind with and inhibit protein coding mRNAs through complementary base pairing. By degrading mRNAs and repressing proteins, miRNAs regulate the cell signaling and cell functions. This paper focuses on innovative mathematical techniques to model gene interactions by algorithmic analysis of microarray data. Our goal was to elucidate which mRNAs were actually degraded or had their translation inhibited by miRNAs belonging to a very large pool of potential miRNAs.

**Results:**

We proposed two chemical kinetics equations (CKEs) to model the interactions between miRNAs, mRNAs and the associated proteins. In order to reduce computational cost, we used a non linear profile clustering method named minimal net clustering and efficiently condensed the large set of expression profiles observed in our microarray data sets. We determined unknown parameters of the CKE models by minimizing the discrepancy between model prediction and data, using our own fast non linear optimization algorithm. We then retained only the CKE models for which the optimized fit to microarray data is of high quality and validated multiple miRNA-mRNA pairs.

**Conclusion:**

The implementation of CKE modeling and minimal net clustering reduces drastically the potential set of miRNA-mRNA pairs, with a high gain for further experimental validations. The minimal net clustering also provides good miRNA candidates that have similar regulatory roles.

## Background

Transcriptional and translational processes are fundamental cell mechanisms, involving three main molecular species: messenger RNA (mRNA) and their associated proteins, as well as microRNAs (miRNAs).

The miRNAs are small non-coding RNAs of roughly 22 nucleotides in length, which can bind with and inhibit protein coding mRNAs through complementary base pairing. A given miRNA can potentially bind and silence hundreds of mRNAs across a number of signaling pathways. These repressive miRNA-mRNA interactions occur in multiple cellular processes [1-3], and involve two distinct modalities: they may directly degrade their target mRNAs, or more often inhibit their translation [4-9].

The best characterized features determining the targets of a specific miRNA are the conserved Watson-Crick pairing to the 5’ region (positions 2–7) of the miRNA, which are the so-called “seed pairing rules” [3,10-13]. Since seed pairing rules are neither sufficient nor necessary for miRNA-target functions [4,14], they have usually been combined with microarray or proteomic analysis to find potential miRNA-target pairs[15-17]. Classical microarray data analysis relies mostly on massive application of correlation analysis and linear statistical techniques to simultaneously acquired gene expression profiles. Combined with profile thresholding and heat map displays, these techniques provide commonly used clues for qualitative inference.

In [18], Principal Component Analysis and linear correlation had been linked with comparative sequence analysis to study microarray data recorded during mouse stem cells differentiation, and to broadly predict potential miRNA-mRNA interactions.

To go beyond the results of the linear microarray analysis applied on time-course microarray data in [18], we have formalized two basic architectures for repressive miRNA-mRNA interactions: “Transcription Degradation” (TD) and “Translation Inhibition” (TI).

Traditional chemical kinetics equations had been proposed to model the transcriptional and translational processes without involving the interaction of miRNAs [19,20].

We have derived Chemical Kinetics Equations (CKEs) to model the dynamics of TD and TI motifs, in the spirit of [20-30]. The equations are algebraically invariant under affine transformation and allow data condensation to reduce computational cost. We have implemented “minimal net” clustering method, which can control the maximum diameter of the clusters, to condense large data sets of gene expression.

Modeling by nonlinear CKEs involves complicated parameter estimation problems to fit the very large set of expression profiles recorded by microarrays. We have developed innovative fast algorithms dedicated to CKE parameter estimation, by optimization of the quality of fit between model and data.

We validate only the parameterized motifs having a high quality of fit to data. To reach robust conclusions we apply a “parameter parsimony” principle, favoring the models having the smallest number of parameters. And we have also evaluated the robustness of our parameter estimation algorithm by algorithm by direct testing on simulated data. These tests did validate our parameter estimation method is quite robust. This nonlinear approach goes further than well-established analysis based on correlation techniques combined with heat map displays.

## Methods

### Basic interaction architectures

To validate if an miRNA *M* does indeed repress a given gene *G*, we model the chemical interactions of M and G within a small network containing the pair (*M*,*G*). We now sketch two basic interaction architectures and their CKE models.

#### Transcription Degradation Motifs (TD-motifs)

**Figure 1:**
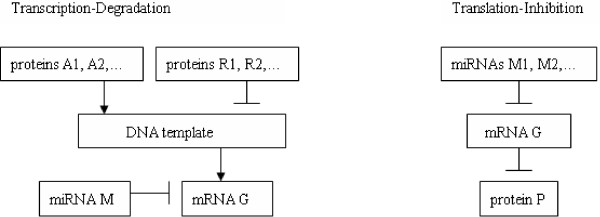
TD-motif and TI-motif.

We call “TD-motif” any interaction architecture, as sketched in Figure 1, involving a single miRNA-mRNA pair (M,G) where G is in Target(M), and *M* degrades the transcription of *G*. The TD motif includes also two sets of proteins rep(G) and act(G), namely the transcriptional “repressors” and “activators” of *G*, denoted by

$$\;\text{rep}(G)=\{R_{1},\ldots R_{k}\}\;\text{and}\;\text{act}(G)=\{A_{1},A_{2},\ldots ,A_{q}\}$$

Let *g*(*t*),*p*(*t*),*m*(*t*),*r*_*i*_(*t*),*a*_*j*_(*t*), be the expression levels at time *t* for the chemical species *G*,*P*,*M*,*R*_*i*_,*A*_*j*_. We model the transcription process by a CKE similar to CKEs proposed in [20,22,25,29,30], but with a complementary term encoding the repressive impact of miRNA *M* on its target mRNA *G*:

(1) $$\frac{\text{dg}(t)}{\text{dt}}=-\mathrm{\beta g}(t)-\text{vg}(t)m(t)+\mathrm{\kappa REP}(t)[\;1-\text{ACT}(t)]$$

where *β*>0 is the degradation rate of *G*, *v*>0 is the reaction rate between *G* and *M*, *κ*>0 is the product of the transcription rate by the concentration *c* of DNA templates, concentration which we assume to be some constant not depending on time (see [20]).

The percentage 0≤*F*(*t*)=*REP*(*t*)[ 1-*ACT*(*t*)]≤1*%* is the fraction of existing DNA templates which are committed at time *t* to transcription of the mRNA gene G. Here the percentages *REP*(*t*) and *ACT*(*t*) are modeled by the following products,

(2) $$\begin{array}{cc} \text{REP}(t) & ={\text{REP}}_{1}(t)\times \ldots \times {\text{REP}}_{q}(t)\\ \text{ACT}(t) & ={\text{ACT}}_{1}(t)\times \ldots \times {\text{ACT}}_{k}(t) \end{array}$$

where

(3) $$\begin{array}{c} {\text{REP}}_{i}(t)=\frac{1}{{\left(1+u_{i}r_{i}(t)\right)}^{{\text{SR}}_{i}}}\\ {\text{ACT}}_{j}(t)=\frac{1}{{\left(1+w_{j}a_{j}(t)\right)}^{{\text{SA}}_{j}}} \end{array}$$

The parameters *SR*_*i*_>0,*u*_*i*_>0 and *SA*_*j*_>0,*w*_*j*_>0 are the number of binding sites and the affinity constant for the transcriptional factors *R*_*i*_ and *A*_*j*_.

Note that the transcription repressors *R*_*i*_ combine multiplicatively their individual impacts *REP*_*i*_ in *REP*(*t*), and that the *REP*_*i*_ are analogous to Hill function (see [19,25]); similar remarks apply to the transcription activators. The multiplicative expressions of *REP*(*t*) and *ACT*(*t*) are typical of a so-called “cis-regulatory” function and have been derived by J. Goutsias [20].

The term *κREP*(*t*)[ 1-*ACT*(*t*)]*dt* is the concentration of new *G* molecules synthesized by transcription during the small time interval [ *t*,*t*+*dt*], while the repressive interactions of M and G eliminates *vg*(*t*)*m*(*t*)*dt* molecules of G, and natural decay destructs *βg*(*t*)*dt* molecules of G.

#### Translation-Inhibition Motifs (TI-motifs)

We call “TI-motif” any interaction architecture, as sketched in Figure 1, involving a set *M*_1_,…,*M*_*r*_ of miRNAs inhibiting the translation of mRNA gene *G*, by repressing the expression of the protein *P* generated by *G*. Let (*p*(*t*),*m*_*i*_(*t*),*g*(*t*)) be the concentrations at time *t* of protein *P*, miRNA *M*_*i*_, and mRNA *G*. In the spirit of [20,21], we model the translation inhibition dynamics by the CKE

(4) $$\frac{\text{dp}}{\text{dt}}=-\mathrm{\gamma p}(t)+\mathrm{\lambda g}(t)H(t)$$

where *γ*>0 and *λ*>0 are the degradation rate and the translation rate for protein *P* and where *H*(*t*) is the percentage of *G* molecules committed at time *t* to the translation of *G*. Thus *H*(*t*) encodes the inhibiting impact of the miRNAs *M*_1_,…,*M*_*k*_ on the translation of *G*, and is modeled as a product of terms similar to *REP*(*t*):

(5) $$\begin{array}{c} \;H(t)\;=H_{1}(t)\times \ldots \times H_{k}(t)\;\text{where}\\ H_{i}(t)\;=\frac{1}{{\left(1+u_{i}m_{i}(t)\right)}^{{\text{SM}}_{i}}} \end{array}$$

The parameters *SM*_*i*_>0 and *u*_*i*_>0 are resp. the number of binding sites and the affinity constant controlling the inhibiting impact of miRNA *M*_*i*_ on the translation of gene *G*. Note that *H*(*t*) decreases when the miRNA concentrations *m*_*i*_(*t*) increase.

Our model for TI motif has been inspired by J. Goutsias [20], and we present below the hypotheses and arguments justifying the expression of *H*(*t*). There is a key difference between the CKEs modeling TD and TI motifs. For TI motifs, the concentration of G-molecules committed to translation is *g*(*t*)*H*(*t*), where *g*(*t*) is the concentration of G-molecules and *H*(*t*)<100*%*.

For TD motifs, the concentration of G-molecules synthesized by transcription is *κF*(*t*)=*αcF*(*t*) with *F*(*t*)<100*%*, where *α* and *c* are respectively the transcription rate and the concentration of DNA templates, assumed to be constant in time.

### Derivation of chemical kinetics equations

Our derivation of the regulation equation for TI motif is quite similar to presentation given for TD motifs in [20], but has several changes in assumptions and formulations. To derive the CKEs (1) and (4), we propose a few hypotheses. 

• Hyp. 1: (TD-motifs) The molecules of the miRNA repressor of gene *G* can strongly bind only at one unique specific site of *G*- molecules, and once a single such strong bind occurs, the corresponding *G* molecule degrades extremely fast.

• Hyp. 2: (TI-motifs) Each miRNA *M*_*j*_ in the set *rep*(*G*) of translation inhibitors of *G* can weakly bind with *G* but only at specific binding sites constituting a set *BIND*_*j*_ of size *S*_*j*_. The sets *BIND*_1_,*BIND*_2_,… are pairwise disjoint. Once a *G* molecule thus binds with one inhibitor *M*_*j*_, then this G molecule will fail to translate.

• Hyp. 3: For any given *G*-molecule, call *X*_*j*_ the random number of sites in *BIND*_*j*_ which actually bind with one “ *M*_*j*_”-molecule. We assume that the random variables *X*_1_,*X*_2_,…. are independent.

Hyp.1 is based on the fact that only a small fraction of all messenger RNAs have more than a single miRNA binding site and miRNA bound with an mRNA gene G has a limited effect on the mRNA G, but affects more substantially the protein P generated by G ([4,31].

Consider first any TI motif involving an mRNA gene *G* generating the protein *P*, as well as a set *rep*(*G*)={*M*_1_,*M*_2_,…*M*_*k*_} of *k* translation inhibitors. We now derive the CKE (4) for this TI motif. Call *p*(*t*),*g*(*t*),*m*_*j*_(*t*) the concentrations of *P*,*G*,*M*_*j*_ and let *H*(*t*) be at time *t* the percentage of existing *G* molecules committed to the translation of *G* into *P*. The basic CKE driving translation of *G* into *P* is then, as seen in [20],

(6) $$\frac{\text{dp}}{\text{dt}}=-\mathrm{\gamma p}(t)+\mathrm{\lambda g}(t)H(t)$$

where *γ*,*λ*, are the degradation and translation rates of *P*.

The main point is to compute *H*(*t*).

For *k*=0, there are no translation inhibitors, and hence *H*(*t*)=100*%*. For *k*=1, let *Q*[ *S*] be the set of *G* molecules with exactly *S* binding sites bound to *M*_1_ molecules, where 0≤*S*≤*S*_1_. Let *q*(*S*,*t*) be the concentration of *Q*[*S*] molecules. We have the forward and backward reactions

(7) $$\begin{array}{c} Q[\;S]+\;M_{1}⇀Q[\;S+1]\;\text{and}\;\;Q[\;S+1]⇀Q[\;S]+M_{1} \end{array}$$

By molecular collision theory [23], the concentration *ρ*(*t*) of free *M*_1_ molecules which bind at time t on *Q*[ *S*]-molecules to produce *Q*[ *S*+1]-molecules by forward reaction 7, is proportional to the product of *m*_1_(*t*)*q*(*S*,*t*) by the number (*S*_1_-*S*) of vacant binding sites. Hence, for some constant *c*,

(8) $$\rho (t)=c(S_{1}-S)m_{1}(t)q(S,t)$$

Similarly the concentration *τ*(*t*) of *M*_1_ molecules freed by backward reaction 7 is given by

(9) $$\tau (t)=\tilde{c}(S+1)q(S+1,t)$$

for some constant $\tilde{c}$. At chemical equilibrium, we have *τ*(*t*)=*ρ*(*t*), and hence

$$q(S+1,t)=um_{1}(t)\frac{S_{1}-S}{S+1}q(S,t)$$

where *u* is a new constant. By recurrence on *S*, this implies

(10) $$\begin{array}{c} q(S,t)=C_{S_{1}}^{S}{\left[\;um_{1}(t)\right]}^{S}q(0,t)\;\text{where}\;\;C_{S_{1}}^{S}=\frac{(S_{1})!}{S!(S_{1}-S)!} \end{array}$$

This entails

(11) $$\begin{array}{c} g(t)=\overset{S_{1}}{\underset{S=0}{\sum }}q(S,t)=q(0,t)\overset{S_{1}}{\underset{S=0}{\sum }}C_{S_{1}}^{S}{\left[\;\text{um}(t)\right]}^{S}\\ \;=\;{\left[\;1+um_{1}(t)\right]}^{S_{1}}q(0,t) \end{array}$$

The set of G-molecules committed to translation into *P* at time *t* is identical to *q*(0,*t*). Thus *H*(*t*)=*q*(0,*t*)/*g*(*t*), and (11) yields $H(t)=1/{\left[\;1+um_{1}(t)\right]}^{S_{1}}$.

Using hypothesis Hyp 3, a recurrence on *k* extends the argument just given for *k*=1, to prove that for *k*≥1, the percentage *H*(*t*) of *G* molecules committed to translation into P is given by:

(12) $$H(t)=\frac{1}{{\left[\;1+u_{1}m_{1}(t)\right]}^{S_{1}}}\times \ldots \times \frac{1}{{\left[\;1+u_{k}m_{k}(t)\right]}^{S_{k}}}$$

which proves CKE (4) for TI motifs.

For TD motifs, the cis-regulation function *F*(*t*) in CKE (1) has been derived in [20]. By using hypothesis 1 and molecular collision theory, we directly deduce that the concentration the concentration of mRNA *G* degraded by miRNA *M* is proportional to the product of both concentrations of *G* and *M*. Thus we justify the miRNA degradation term -*vg*(*t*)*m*(*t*).

### Invariance by affine profile transformations

#### Affine profile transformations

Call *Γ*⊂*R*^*q*^ the set of all possible expression level profiles *r*(*t*) indexed by time *t*_1_,…,*t*_*q*_. To any pair *T*=(*a*,*b*) of real numbers, we associate an *affine profile transformation*

r→Trdefined byr(t)→(ar(t)+b)

for all time dates *t*. Let  be the set of all such affine profile transformations.

In microarray data sets, expression levels of genes are recorded via optical analysis of fluorescence intensities, and hence depend strongly on experimental acquisition modalities. So relative expression levels between pairs of recorded chemical species are more meaningful quantities, and graphic displays of microarray data by “heat maps” often involve logarithms of raw data.

Our microarray data record expression levels which as a first approximation can be viewed as unknown affine transformations of concentrations. Since our CKEs (1), (4) were derived for concentrations, we need to check how these CKEs and their parameters change under generic affine profile transformations.

#### Affine invariance of CKE models

Consider first a TD-motif involving an mRNA gene *G*, a protein *P*, an miRNA *M*, transcription repressive proteins *R*_1_,*R*_2_,…, and transcription activating proteins *A*_1_,*A*_2_,…. The CKE model (1) links the concentrations *g*,*p*,*m*,*r*_*i*_,*a*_*j*_ of *G*,*M*,*P*,*R*_*i*_,*A*_*j*_. Let $ĝ,\hat{p},\hat{m},{\hat{r}}_{i},{â}_{j}$ be the corresponding recorded expression profiles. Assume that each such recorded profiles $\hat{r}$ is linked to the concentration profile 4 by some *unknown* affine profile transformation *T*_*r*_.

From CKE (1), one directly deduces that $ĝ,\hat{p},\hat{m},{\hat{r}}_{i},{â}_{j}$ verify a new CKE having an algebraic form completely similar to CKE (1), but where the original parameters *β*,*v*,*κ*,*u*_*i*_,*w*_*j*_ are replaced by new parameters $\hat{\beta },\hat{v},\hat{\kappa },{û}_{i},{ŵ}_{j}$, and where the integers *SR*_1_,*SR*_2_,…, and *SA*_1_,*SA*_2_,…, remain unchanged.

The new parameters are easily expressed in terms of the original ones and of the coefficients of the affine transformations, but this is irrelevant practically since we will use microarray data to directly compute the new parameters for a CKE of type (1) linking recorded expression levels.

Similar computations for TI-motifs show that this affine invariance property also holds for CKE (4). Hence to model a TD or a TI motif, we can fit a CKE model of type (1) or (4) to recorded expression profiles, even though the theoretical model justification involved true concentrations, which are not directly measured by microarrays.

The key assumption is that, for each chemical species C, the expression levels ĉ(t) of C recorded by microarray are approximately linked to the concentration *c*(*t*) of C by some affine relation ĉ(t)=a(C)c(t)+b(C), where the unknown coefficients *a*(*C*) and *b*(*C*) may depend on the species C.

The preceding algebraic model invariance under multiple affine profile transformations strongly suggests that *adequate distances between dynamic profiles of recorded expression levels should be invariant under affine profiles transformations*, as developed in the next section.

### Condensation of expression levels profiles

Microarray data typically record several tens of thousands gene expression profiles. So computational costs to fit microarray data to all potential TD-motifs and TI-motifs would of course be prohibitive. A natural option to reduce combinatorial explosion is to cluster the observed profiles.

Since our goal is to model expression profiles by ODEs of type (1) and (4), we need to control the diameters of all clusters of expression profiles. This led us to reject hierarchical clustering as well as K-means clustering, and to implement in the space of expression profiles a “minimal-net” clustering technique, inspired by an innovative technique for automatic generation of prototypes in shape spaces (see [32]). In view of the preceding section, the diameters of clusters in the space of profiles should be measured by a distance invariant under affine profiles transformations.

#### Affine invariant distance between profiles

Recall that *Γ*⊂*R*^*q*^ is the set of all profiles *r*(*t*) indexed by time dates *t*_1_,…,*t*_*q*_. The mean and variance of a profile *r* are denoted by

$$\begin{array}{ll} \;\bar{r}=\frac{1}{q}[\;r(t_{1})+\ldots +r(t_{q})]\;;\; & \;\\ \text{var}(r)=\frac{1}{q}[\;{\left(r(t_{1})-\bar{r}\right)}^{2}+\ldots +{\left(r(t_{q})-\bar{r}\right)}^{2}]\; & \; \end{array}$$

Define normalized profiles *nor*(*r*) and profiles correlations *corr*(*r*_1_,*r*_2_) by

$$\begin{array}{ll} \;\text{nor}(r)(t)=\frac{r(t)-\bar{r}}{\sqrt{\text{var}(r)}}\;;\; & \;\\ \text{corr}(r_{1},r_{2})=<\text{nor}(r_{1}),\text{nor}(r_{2})>\; & \; \end{array}$$

where <·,·> is the usual scalar product in *R*^*p*^.

We then define a *distanceD*(*r*_1_,*r*_2_) between profiles *r*_1_ and *r*_2_ by

$$D(r_{1},r_{2})=\sqrt{2-2\;\text{corr}(r_{1},r_{2}))}$$

For any affine profile transformation T in , one has *nor*(*r*)=*nor*(*Tr*), and hence *D*(*r*_1_,*r*_2_)=*D*(*nor*(*r*_1_),*nor*(*r*_2_). Thus for any affine profile transformations *T*_1_,*T*_2_, and any profiles *r*_1_,*r*_2_ one has

$$D(T_{1}r_{1},T_{2}r_{2})=D(r_{1},r_{2})$$

So the profile distance D *is invariant by affine profile transformations*

#### Minimal net clustering

The set *Γ* of all profiles is now endowed with a distance *D* invariant by affine profile transformations. Call *mPR* the large set of miRNA profiles recorded at time *t*_1_,…,*t*_*q*_ by our microarrays. We fix a maximum radius *ε* for profiles clusters. We seek to partition *mPR* into disjoint clusters *CL*_1_,…,*CL*_*r*_ such that each *CL*_*j*_ has diameter inferior to 2*ε*, and we also want the number *NN* of clusters to be as small as possible. We have implemented an iterative algorithm to generate this type of minimal net clustering, in the spirit of [32].

Define a distance function *D*(*x*,*y*), where *x*,*y* represent the observations of the data. Denote by *D*(*x*,*Y*) the distance between an observation *x* and a set *Y*, where *D*(*x*,*Y*)= min*y*∈*YD*(*x*,*y*). Let Let [ *x*] denote the cluster containing single point *x*, and *Γ* be the set of all observations, the minimal net algorithm is as follows: 

1. step 1, let (*x*_1_,*y*_1_)=*argmax*_*x*,*y*_*D*(*x*,*y*). Then let *x*_1_ and *y*_1_ become the representatives of two initial clusters. Let *CL*_1_= [ *x*_1_],*CL*_2_= [ *x*_2_], *C*_1_={*CL*_1_,*CL*_2_} and *R*_1_=*Γ*∖*C*_1_ representing the remaining points in *Γ* excluding the 2 clusters.

2. After step *n*-1, we obtain *C*_*n*-1_={*CL*_1_,*CL*_2_,…*CL*_*n*_} and *R*_*n*-1_=*Γ*∖*C*_*n*-1_, where *CL*_*j*_ is a cluster that has single point, *j*=1,…,*n*+1.

In step *n*, let $\text{HD}=\underset{x\in R_{n-1}}{\max}D(x,C_{n-1})$, representing the maximum distance between observation *x* in *R*_*n*-1_ and set *C*_*n*-1_. If *HD*>*ε*, find $x_{\text{HD}}={\text{argmax}}_{x\in R_{n-1}}D(x,C_{n-1})$. Let *C*_*n*_={*C*_*n*-1_,*x*_*H* *D*_}, *R*_*n*_=*Γ*∖*C*_*n*_. Repeat until *HD*≤*ε*.

3. Assume the loop stop at step *NN*, we have *C*_*N* *N*_={*CL*_1_,*CL*_2_,…*CL*_*N* *N*+1_}. For an observation *x*, find the point $y_{{\text{CL}}_{k}}$, belonging to cluster *CL*_*k*_ in *C*_*N* *N*_, that is closest to *x*, i.e. $y_{{\text{CL}}_{k}}={\text{argmin}}_{y\in C_{\text{NN}}}(D(x,C_{\text{NN}}))$, then assign *x* to the cluster *CL*_*k*_.

We apply this minimal net clustering algorithm to the set of all miRNA profiles recorded by our microarrays. We define the cluster diameter *diam*(*CL*) of cluster CL as the maximum distance of any two observations in the cluster, i.e. *diam*(*CL*)= max*x*,*y*∈*CLD*(*x*,*y*), where. Compared with the commonly used clustering method such as K-means and Hierarchical clustering, the minimal net algorithm allows us to control the diameter of all clusters by setting the threshold *ε* representing the supremum radius of the cluster, while the K-means and hierarchical clustering is often used to determine the number of of clusters but not their diameters. If we select a very small threshold *ε*, the expression levels of genes in the same cluster can be considered almost identical.

### Parameter estimation for the CKE models

#### Parameters estimation strategy

A generic strategy for parameter estimation in CKEs systems is to minimize a cost function evaluating the discrepancy between model predictions and experimental data. Each one of our CKE models has a single output variable, namely the expression level *g*(*t*) of mRNA gene G for a TD motif, and the expression level *p*(*t*) of protein P for a TI motif. The output variable *g*(*t*) or *p*(*t*) of a CKE model can be estimated by a function ĝ(t) or $\hat{p}(t)$ once one knows the profiles of all other molecular species involved in the model. The estimators ĝ(t) or $\hat{p}(t)$ are respectively determined by CKE (1) or CKE (4).

Each CKE models is parameterized by a parameter vector **w** of dimension *n*_*p* *a* *r*_. The quality of fit of this model with recorded profiles data is quantified by the size *ERR*(**w**) of the estimation error defined as follows

$$\text{ERR}(\text{w})=\underset{t}{\max}|f(t)-\hat{f}(t)|$$

where the output variable *f*(*t*) is equal to *g*(*t*) for TD motifs and to *p*(*t*) for TI motifs. The concrete goal is to find the best parameter vector **w** by minimization of the lack of fit *ERR*(**w**) over all possible values of *w*.

There are no magic solutions for such non linear minimization problems. Moreover fast computing was essential here, since we usually have to solve a very large number of similar “quality of fit maximization” problems when dealing with large microarray datasets.

We have tested several generic cost minimization approaches (see [33-35]) such as “genetic algorithms”, as well as “gradient descent” to minimize a sum of squared modeling residuals. These two techniques turned out to require far too much computing time and were often unreliable due to their high dependence on initialization values.

We hence developed our own fast CKE parametrization algorithms to optimize the quality of fit between CKE models and microarray profiles data. This optimized quality of fit, adequately balanced by a systematic emphasis on parsimoniously parameterized models, becomes an essential clue to decide which potential interactions one should validate between miRNAs, mRNAs, and associated proteins.

#### Parameters parsimony requirement

Robustness of CKE parametrization is the main motivation for our parameter parsimony requirements. Consider CKE (1) modeling a TD-motif *TDM* involving *n*_*a* *c* *t*_ activators and *n*_*r* *e* *p*_ repressors for the transcription of mRNA gene *G*, and one miRNA *M* degrading the transcription of *G*. Then the number *n*_*p* *a* *r*_ of unknown parameters is *n*_*p* *a* *r*_=3+2(*n*_*a* *c* *t*_+*n*_*r* *e* *p*_). Each profile *g*(*t*),*m*(*t*),*a*_*i*_(*t*),*r*_*j*_(*t*) is recorded at *t*=*t*_1_,…,*t*_*p*_. The CKE outputs for *TDM* are *g*(*t*_1_),…,*g*(*t*_*p*_). Hence each microarray data set provides (*p*-1) equations linking the *n*_*p* *a* *r*_ unknown parameters, since the recorded *g*(*t*) should be very close to the predicted values ĝ(t) obtained by solving the ODE (1) with initial value *g*(*t*_1_).

Vapnik’s results on model fitting (see [36]) show that robust accuracy of parameter estimates requires fairly high values of the ratio of (*p*-1)/*n*_*p* *a* *r*_. So *n*_*p* *a* *r*_<<(*p*-1) is a necessary constraint, and we will impose the *parameter parsimony requirementn*_*p* *a* *r*_≤(*p*-1)/4. Indeed when *n*_*p* *a* *r*_>(*t*_*p*_-1), CKE models are overfitted and parameters are poorly estimated.

### CKE Parameter estimation

#### TI-motifs: plausible ranges for parameters

Consider a generic TI motif *TIM* involving an mRNA gene *G*, its associated protein *P*, and *k* translation inhibiting miRNAs [ *M*_1_,…,*M*_*k*_]. Let *p*(*t*),*g*(*t*),*m*_*i*_(*t*) be the expression levels of *P*,*G*,*M*_*i*_. We want to model *TIM* by CKE (4).

Parametrized by the vector

$$\text{w}=\;[\;\gamma ,\lambda ,u_{1},S_{1},\ldots ,u_{k},S_{k}]$$

which involves (2*k*+2)≤8 parameters.

The degradation and translation rates *γ* and *λ* of protein *P* were unknown for most proteins.

According to results from [31], only a small percentage 2% of our 30,000 mRNAs have more than 2 potential binding sites for miRNAs, and only 0.02*%* mRNAs have as many as 7 such binding sites. So in our parameter estimations it is reasonable to restrict the number of binding sites *S*_*j*_ for miRNA *M*_*j*_ to be at most 5.

#### TI-motifs: optimizing the quality of fit

The inputs of CKE (4) are the initial value *p*(*t*_1_) and the expression levels *g*(*t*),*m*_1_(*t*),…,*m*_*k*_(*t*) recorded at time dates *t*_1_,…,*t*_*q*_.

Discretizing the ODE (4) at time *t*_1_,…,*t*_*q*_, we get

(13) $$p(t_{j+1})-p(t_{j})=-\mathrm{\gamma p}(t_{j})+\mathrm{\lambda g}(t_{j})H(t_{j})$$

where the percentage H(t) is as recalled above. By summation this implies, for *j*=2,3,.…,*q*-1, the relation

(14) $$p(t_{j+1})-p(t_{1})=-\mathrm{\gamma Q}(t_{j})+\mathrm{\lambda L}(t_{j})$$

where

$$Q\left(t_{j}\right)=\overset{j}{\underset{n=1}{\sum }}g\left(t_{n}\right)\;;\;L\left(t_{j}\right)={\text{sum}}_{n=1}^{j}g\left(t_{n}\right)H\left(t_{n}\right)$$

In view of equation (14), when all expression profiles involved have been recorded until time *t*_*j*_, one can predict the still unknown value of *p*(*t*_*j*+1_) by the following natural estimator $\hat{p}(t_{j+1})$,

(15) $$\hat{p}(t_{j+1})=p(t_{1})-\mathrm{\gamma Q}(t_{j})+\mathrm{\lambda L}(t_{j})$$

The quality of fit of this CKE model with recorded profiles data will be quantified by *the sizeERR*(**w***) of the estimation error* numerically computed as follows

(16) $$\text{ERR}(w)=\underset{j=1,\ldots ,q}{\max}\;|\;p(t_{j})-\hat{p}(t_{j})\;|$$

which in view of (15) can be reformulated as

(17) $$\text{ERR}(w)=\underset{j=1,\ldots ,T}{\max}\;|\;\pi_{j}+\mu_{j}\gamma -\nu_{j}\lambda \;|$$

where for *j*=1,…,*q* we have set

(18) $$\pi_{j}=p(t_{j})-p(t_{1})\mu_{j}=Q(t_{j})\nu_{j}=L(t_{j})$$

We seek a parameter vector **w** minimizing the cost function *ERR*(**w**) in the parametric domain defined by

$$\gamma >0\;;\;\lambda >0\;;\;u_{i}>0\;;\;1\leq S_{i}\leq 5$$

#### TI-motif: parameter estimation algorithm

For each *i*=1…*k*, fix an arbitrary integer 1≤*S*_*i*_≤5. Call ${\bar{m}}_{i}$ and ${\bar{H}}_{i}$ the respective medians over time *t* of the functions *m*_*i*_(*t*) and *H*_*i*_(*t*). Since the function $H_{i}(t)=1/{\left[\;1+u_{i}m_{i}(t)\right]}^{S_{i}}$ is monotonous in *m*_*i*_(*t*), we have

(19) $${\bar{H}}_{i}=1/{\left[\;1+u_{i}{\bar{m}}_{i}\right]}^{S_{i}}$$

The assumption 0<*H*_*i*_(*t*)<1 yields $0<{\bar{H}}_{i}(t)<1$. We will discretize the possible values of ${\bar{H}}_{i}(t)$ by constraining them to belong to a grid *h*_1_,…,*h*_*s*_ of 1≤*s*≤99 percentage values equally spaced in [ 0,1]. Since ${\bar{m}}_{i}$ is known, we invert equation (19) to compute a corresponding grid *GRD*_*i*_ of *s* potential values for $u_{i}=\frac{1}{{\bar{m}}_{i}}({\left(\frac{1}{{\bar{H}}_{i}}\right)}^{1/S_{i}}-1)$.

Now for 1≤*i*≤*k*, select and fix arbitrary values *u*_*i*_ in the grid *GRD*_*i*_. After selecting as restrictive above the set of parameter vector *U*= [ *u*_1_,*S*_1_,…*u*_*k*_,*S*_*k*_], the function *H*(*t*) is then completely determined for all *t*.

Note that the set  of all possible such choices for *U* is of cardinal inferior to *N*=(5*s*)^*k*^. Since we do explore each one of these possibilities separately, we need to keep the number *N* at a reasonable level, so we selected *s*=99 for *k*=1, *s*=60 for *k*=2, *s*=20 for *k*=3 etc.

Fix any *U* in . Since all recorded profiles involved in the TD motif are available at time *t*_1_,…,*t*_*q*_, we can use the *U* value to directly compute all the values *H*(*t*_*j*_), and then all the numbers *π*_*j*_,*μ*_*j*_,*ν*_*j*_ defined by (18). We want to find values of the two last parameters *γ*>0 and *λ*>0 which will minimize

$$\;\text{ERR}(\gamma ,\lambda )=\underset{j=1,\ldots ,q}{\max}\;|\;\pi_{j}+\mu_{j}\gamma -\nu_{j}\lambda \;|$$

This problem is equivalent to minimizing the linear objective function:

Ψ(γ,λ,z)=z

under the (2*q*+3) linear inequality constraints

$$\begin{array}{c} \;\gamma >0\;;\;\lambda >0\;;\;z>0\\ z-(\pi_{j}+\mu_{j}\gamma -\nu_{j}\lambda )\geq 0\;\text{for}\;j=1,\ldots ,q\\ z+(\pi_{j}+\mu_{j}\gamma -\nu_{j}\lambda )\geq 0\;\text{for}\;j=1,\ldots ,q \end{array}$$

This is a classical *constrained linear programming* problem, which can be solved by well known fast linear programming algorithms [37], to provide the optimal values *γ*^∗^,*λ*^∗^,*z*^∗^. Then *z*^∗^=*ERR*(*γ*^∗^,*λ*^∗^) is the minimal value of *ERR*(*γ*,*λ*).

These optimal values are functions *γ*^∗^(*U*),*λ*^∗^(*U*),*z*^∗^(*U*) of the partial vector of parameters *U* in . We then select the optimal **U**^∗^ in  as the value of *U* which minimizes *z*^∗^(*U*) over . The optimal parametrization **w**^∗^ of our TD motif is then given by **w**^∗^= [ *γ*^∗^(*U*^∗^),*λ*^∗^(*U*^∗^),*U*^∗^]. This new parametrization algorithm is fairly fast and has good accuracy. On a current laptop PC, our non-optimized MATLAB code implementation required less than 5 minutes of CPU time for the parametrization of a typical TI-model with 38 time points and 9 parameters [38]. After code optimization and a re-implementation in C, we expect this CPU time to be reduced to 2 minutes. The algorithm does not require any knowledge of the parameters ranges except for the number of binding sites *S*, which is an advantage for the range of reaction rates of of many molecules of interest are usually unknown.

#### TD-motifs: parameter estimation algorithm

The parametrization algorithms just presented also apply to TD models, as we now sketch. The output variable is now the expression level *g*(*t*) of mRNA gene G.

Discretize the ODE (1) at time dates *t*_1_,…,*t*_*q*_, to get

(20) $$g(t_{j+1})-g(t_{j})=-\mathrm{\beta g}(t_{j})-\text{vg}(t_{j})m(t_{j})+\mathrm{\kappa F}(t_{j})$$

where F(t) is defined in (1). By summation this implies the relations

(21) $$g(t_{j+1})-g(t_{1})=-\mathrm{\beta B}(t_{j})-\text{vV}(t_{j})+\mathrm{\kappa K}(t_{j})$$

where

$$\begin{array}{ll} B(t_{j})=\overset{j}{\underset{n=1}{\sum }}\;g(t_{n})\;;\;V(t_{j})=\overset{j}{\underset{n=1}{\sum }}\;g(t_{n})m(t_{n})\;;\; & \;\\ K(t_{j})={\text{sum}}_{n=1}^{j}\;F(t_{n})\; & \; \end{array}$$

When all expression profiles involved have been recorded until time *t*_*j*_, one can predict the unknown value of *g*(*t*_*j*+1_) by the estimator $ĝ(t_{j+1})$,

(22) $$ĝ(t_{j+1})=g(t_{1})-\mathrm{\beta B}(t_{j})-\text{vV}(t_{j})+\mathrm{\kappa K}(t_{j})$$

The quality of fit of this CKE model with the recorded profiles data is quantified by *the size of the prediction error* which is a function *ERR*(**w**) of the parameter vector **w**

$$\text{ERR}\left(w\right)=\underset{j=1,\ldots ,q}{\max}\;|\;g\left(t_{j}\right)-\hat{g}\left(t_{j}\right)\;|$$

so that

(23) $$\text{ERR}\left(w\right)=\underset{j=1,\ldots ,q}{\max}\;|\;\tau_{j}+\rho_{j}\beta +\eta_{j}v-\theta_{j}\kappa \;|$$

where we have set

$$\tau_{j}=g(t_{j})-g(t_{1})\;\rho_{j}=B(t_{j})\;\eta_{j}=V(t_{j})\;\theta_{j}=K(t_{j})$$

The TD model parameters *SR*_*i*_>0,*u*_*i*_>0 and *SA*_*j*_>0,*w*_*j*_>0 are the number of binding sites and the affinity constants for the transcriptional factors *R*_*i*_ and *A*_*j*_. They constitute a partial parameter vector *U* which we restrict as above by first imposing a moderate upper bound *S*_*m* *a* *x*_ on all the integers *SR*_*i*_,*SA*_*j*_. and by selecting, also as done above, adequate finite grids for the values of the *u*_*i*_ and the *w*_*j*_. This constrains *U* to belong to a finite set . The cardinal N of  is forced to remain at most of order 10^5^, by adequate constraints on *S*_*m* *a* *x*_ and on the coarseness of the *u*_*i*_ grids and the *v*_*j*_ grids.

To minimize *ERR*(**w**), we fix as above an arbitrary *U* in . We can then compute all the numbers *τ*_*j*_,*ρ*_*j*_,*η*_*j*_,*θ*_*j*_. Since *U* is fixed, the error size *ERR*(**w**) becomes a function *E*(*β*,*v*,*κ*) of the last 3 positive parameters (*β*,*v*,*κ*), still given by equation (23). As above the minimization of *E*(*β*,*v*,*κ*) is equivalent to a constraint linear programming problem where we want to minimize the linear function *Φ*(*β*,*v*,*κ*,*z*)=*z* over the following set of (2*q*+4) linear inequalities

$$\begin{array}{cc} \;\beta >0\;;\;v>0\;;\;\kappa >0\;;\;z>0\\ z-(\tau_{j}+\rho_{j}\beta +\eta_{j}v-\theta_{j}\kappa ) & \geq 0\;\text{for}\;j=1,\ldots ,q\\ z+(\tau_{j}+\rho_{j}\beta +\eta_{j}v-\theta_{j}\kappa ) & \geq 0\;\text{for}\;j=1,\ldots ,q \end{array}$$

Solving this constraint linear programming problem generates optimal parameters (*β*^∗^,*v*^∗^,*κ*^∗^) and a minimal error *z*^∗^, which are all functions of *U*. One concludes as above by selecting an optimal *U*^∗^ minimizing *z*^∗^(*U*) over all *U* in .

### Quality of fit for CKE models

Consider any TD motif or TI motif . We have seen how to compute a parameter vector **w**^∗^ optimizing the quality of fit between microarray data and our CKE model for . This was done by minimizing $\text{ERR}(w)=\underset{t}{\max}\;|\;f(t)-\hat{f}(t)\;|$, where *f*(*t*) is the main output variable of , and $\hat{f}(t)$ is the estimation of *f*(*t*) based on the CKE parametrized by ***w***.

For the optimal CKE parametrization **w**^∗^, the lack of fit to data can then be evaluated by *ERR*(**w**^∗^). However we have seen in section ‘Invariance by affine profile transformations’ that when comparing two expression profiles recorded by microarray, natural distances between profiles should be roughly invariant by changes of scale for these profiles. It would then be tempting to replace the absolute error of estimation $\left|\;f(t)-\hat{f}(t)\;\right|$ at time *t* by the relative error of estimation $\frac{\left|f(t)-\hat{f}(t)\right|}{f(t)}$. But relative errors become quite large whenever the output profile *f*((*t*) is close to zero. To avoid such spuriously large error values, while still preserving scale invariance whenever *f*(*t*) is not close to zero, we define the Smoothed Relative Error of estimation *SRE*(*t*) at time *t* by the following formula, where $\bar{f}$ denotes the mean value of the profile *f*,

(24) $$\text{SRE}(t)=\frac{\left|f(t)-\hat{f}(t)\right|}{f(t)}\;\text{when}\;\;f(t)>0.15\;\bar{f}$$

(25) $$\text{SRE}(t)=\frac{\left|f(t)-\hat{f}(t)\right|}{\bar{f}}\;\text{when}\;\;f(t)\leq 0.15\;\bar{f}$$

We finally quantify the *Modeling ErrorMODER* for the optimally parametrized CKE model of motif  by

$$\text{MODER}=\underset{t}{\max}\text{SRE}(t)$$

## Results and discussion

### Examples of application

We implemented our model of TI on microarray data of mouse stem cells undergoing RA-induced differentiation, as provided by LC Science Inc, and previously analyzed by classical techniques in [18]. We took the recorded expression profiles for proteins/mRNAs GCNF, Oct4, Nanog and Sox2 at time points (0, 1.5, 3, 6)/(0, 3, 6) and expression levels for 266 miRNAs on days 0, 1, 3, 6 from [38] during ES cell differentiation. These profile data were interpolated at 19 intermediary time points, by Piecewise Cubic Hermite Interpolation (PCHIP) and the number of parameters were limited to be 4, i.e. only 1 upstream miRNA was selected for the model, to satisfy the parameter parsimony requirement.

For miRNA *M*_*i*_, the following linear transformation, which could be viewed as normalization, was done:

$$\hat{m_{i}}\left(t\right)=\frac{m_{i}\left(t\right)-\bar{m_{i}}\left(t\right)}{\sigma \left(m_{i}\right)}+1$$

where $\sigma (m_{i})=\sqrt{\underset{t}{\sum }{\left(m_{i}(t)-\bar{m_{i}}(t)\right)}^{2}}$, *t*=0,1/3,…,6. Since $∥\frac{m_{i}(t)-\bar{m_{i}}(t)}{\sigma (m_{i})}∥\leq 1$, $\hat{m_{i}}(t)$ is positive for *t*=0,1/3,…,6. Taking *ε*=0.15, we applied Minimal Net clustering (the MATLAB code can be downloaded through Additional file 1) to the transformed data of miRNAs, $\hat{m_{i}}(t),i=1,2,\ldots ,266$, and obtained 107 clusters, in which the maximum cluster contains 14 miRNAs. We consider that the miRNAs belonging to the same cluster share the same normalized expression level within a negligibly small error. For each cluster *CL*_*j*_, *j*=1,…,107, we determine the miRNA *MC*_*j*_ which is the representative of the cluster *CL*_*j*_ for the distance *D*, and we let *mc*_*j*_(*t*) be the expression level of *MC*_*j*_ at time *t*. We call *mc*_*j*_(*t*) is the expression level of cluster *CL*_*j*_.

**Figure 2:**
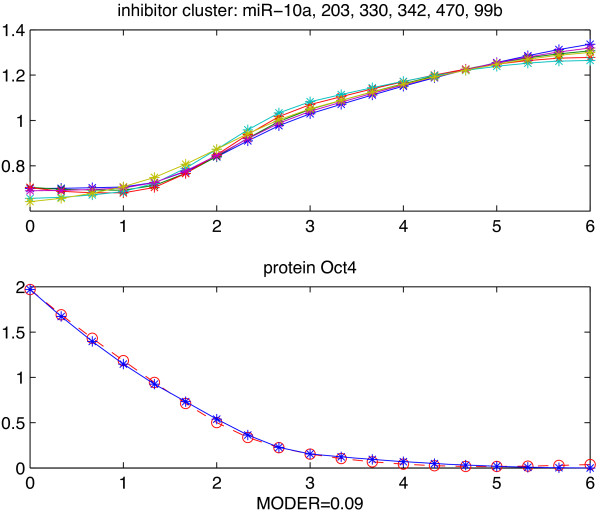
**Example of validated TI motif.** Example of TI motif repressing Oct4. All expression profiles are over days 0–6. Upper 6 profiles: normalized expression levels of numerically validated miRNAs in the same cluster: mmu-miR-10a, 203, 330, 342, 470 and 99b. Bottom 2 profiles: Blue line = recorded levels. Red dash line = predicted levels. “MODER” is the model global relative error of prediction.

We successively implemented the TI model with only one repressor miRNA (the MATLAB code can be downloaded through Additional file 2). After parametric modeling of our pre-selected 107 TI motifs, and evaluation of their quality of fit, we have validated only 3 clusters of miRNAs as translational inhibitors repressing protein Oct4. If an miRNA in *CL*_*j*_ is validated by modeling as a potential repressor, all other miRNAs belonging to *CL*_*j*_ are also potential repressors and can be validated numerically as well by the form invariability of the model under affine transformation. Here we present one of these three validated miRNAs of Oct4 (see Figure 2), where the centroid of the cluster is miRNA mmu-miR-10a, while the other 6 miRNAs in the cluster are mmu-miR-203, 330, 342, 470 and 99b. We used TarBase [39] to search all the experimentally validated miRNAs targeting Oct4 (Pou5f1) in Mus musculus. By TarBase, only mmu-miR-470 has been experimentally validated and it is also numerically validated by our TI model.

**Figure 3:**
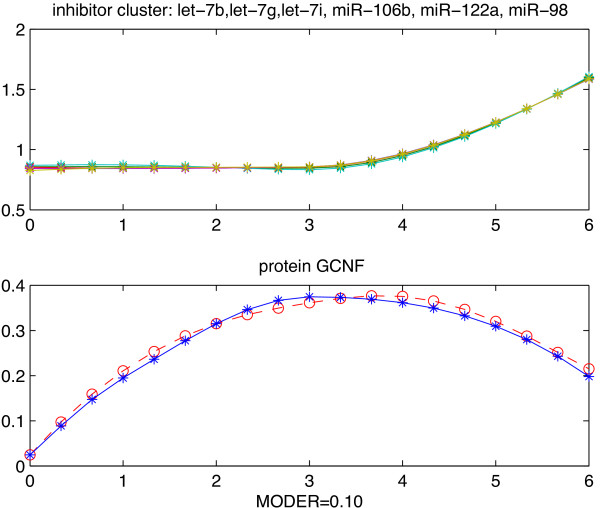
**Example of validated TI motif.** Example of TI motif repressing GCNF. All expression profiles are over days 0–6. Upper 6 profiles: normalized expression levels of numerically validated miRNAs in the same cluster: mmu-let-7b, let-7g, let-7i, miR-106b, miR-122a, miR-98. Bottom 2 profiles: Blue line = recorded levels. Red dash line = predicted levels. “MODER” is the model global relative error of prediction.

For protein GCNF, only two miRNAs, mmu-let-7b and mmu-let-181a, have been experimentally validated by TarBase and both of them belong to the list of the 20 miRNAs numerically validated by our TI modeling (we presented the validated cluster containing mmu-let-7b in Figure 3). Our modeling approach did not validate any miRNA repressing both proteins Nanog and Sox2, while there are 3 miRNAs and 1 miRNA experimentally validated as separate repressors of Nanog and Sox2 respectively.

**Figure 4:**
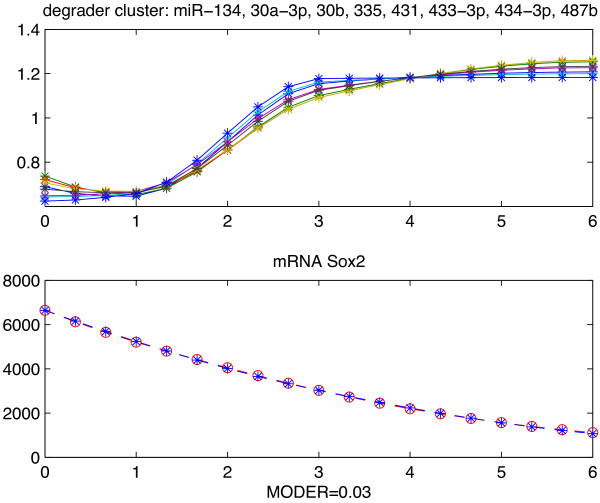
**Example of validated TD motif.** Example of TD motif repressing Sox2. All expression profiles are over days 0–6. Upper 8 profiles: normalized expression levels of numerically validated miRNAs in the same cluster: mmu-miR-134, 30a-3p, 30b, 335, 431, 433-3p, 434-3p, 487b. Bottom 2 profiles: Blue line = recorded levels. Red dash line = predicted levels. “MODER” is the model global relative error of prediction.

We here also present one example of TD motif for downstream factor mRNA Sox2 (Figure 4). With the assumption that the transcription factors are proteins Oct4 and Nanog [38], we validated cluster mmu-miR-134, 30a-3p, 30b, 335, 431, 433-3p, 434-3p, and 487b as degraders. Although (mmu-miR-134, Sox2) is also an experimentally tested pair, we will not discuss deep in detail the validation results of the TD motifs in this paper. The main reason is that the validation results of TD motifs depends much on our knowledge of the transcription factors. More discussion is in the subsection below.

### Discussion

In [38], we pre-selected the potential miRNAs for each gene/protein by TargetScan 5.0 and miRanda before applying the two models. The pre-selection of miRNA candidates were not necessary though it greatly reduced the computational cost. However, the application of the CKE modeling was dependant on the target prediction algorithm, such as TargetScan or miRanda. Therefore, we introduced Minimal Net Clustering in this paper so that the data was condensed and the computational cost could be reduced by a purely numerical method without biological bias.

Since the results of TD model depend on information of transcription factors, the modeling validates not only miRNAs acting as mRNA degraders but also upstream transcription factors simultaneously. In [38] we numerically validated proteins GCNF, Oct4 and Nanog as transcription factors for mRNAs Oct4 and Nanog, while Marson et al. and Boyer et al. [40,41] claimed that Oct4 and Sox2 are bounded and act together with Nanog as transcription factors. Considering the impact of the transcription factors, the TD modeling may be less convincing unless the transcription factors are fixed as experimentally validated. In this paper we presented more details on the implementation and validation results of the TI model in order to focus on the validation of miRNAs and avoid the influence of assumptions of transcription factors.

TarBase shows that, for protein GCNF the experimentally validated miRNAs are mmu-let-7b and mmu-miR-181a; for protein Oct4 the experimentally validated miRNA is mmu-miR-470; for protein Nanog the experimentally validated miRNAs are mmu-miR-134, mmu-miR-470, and mmu-miR-296; for protein Sox2 the experimentally validated miRNA is mmu-miR-134. In this paper, we have validated by TI modeling and minimal net clustering all the experimentally tested miRNA repressors of GCNF and Oct4. In our previous work [38], actually none of these miRNA repressors had yet been studied for modeling for the four proteins because of the restriction of pre-selection. And only the pair (mmu-miR-181a, GCNF) was validated by the classical correlation analysis done in Gu et al. [18] for the 4 proteins GCNF, Oct4, Nanog, Sox2. Therefore, the TI modeling combined with data condensation not only reduced computational cost but also clearly extended the set of miRNA inhibitors validated by model fitting to microarray data.

Since each numerically validated miRNA cluster may contain two or more miRNAs, the miRNAs in the same cluster could also be considered as potential candidate inhibitors for further experiments to validate. For instance, as Figure 2 shows, mmu-miR-203, 330, 342, 10a and 99b are potential candidates for protein Oct4 for they are in the same cluster as mmu-miR-470, which is validated by both the numerical modeling and experiments.

**Figure 5:**
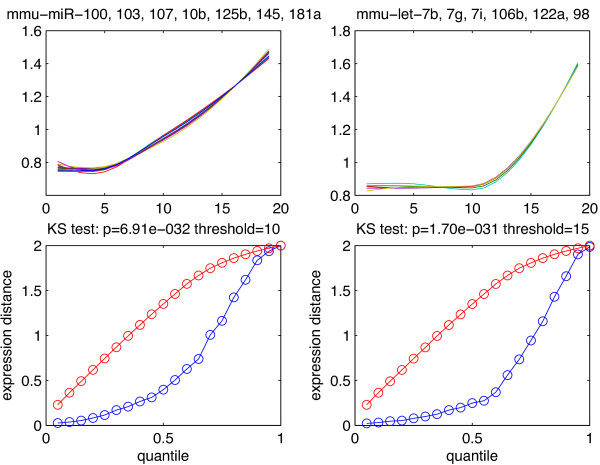
**miRNA clusters and analysis of mature sequences and expression levels.** Top left, expression profiles for a cluster of 7 miRNAs. Top right, expression profiles for a cluster of 6 miRNAs. Bottom left, the blue curve is the quantile curve of expression distances for miRNA pairs with high alignment scores (*NWA*>10), the red curve is the analogous quantile curve for miRNA pairs with low alignment scores (*NWA*≤10). Note that the blue curve lies below the red curve indicates that when NWA increases, then expression distance tends to stochastically decrease. Bottom right, similar to bottom left but the NWA threshold changes to *NWA*=15.

After the pair (mmu-miR-181a, GCNF) was well validated by our CKE model, we found that the cluster (see Figure 5, top left) containing mmu-miR-181a also includes mmu-miR-103 and mmu-miR-107, which are two known miRNAs that have the same roles in regulating insulin sensitivity and promoting metastasis of colorectal cancer [42,43]. We also checked that miR-103 and miR-107 have almost the same mature sequences: 

• mmu-miR-103: AGCAGCAUUGUACAGGGCUAUGA

• mmu-miR-107: AGCAGCAUUGUACAGGGCUAUCA

After the pair (mmu-let-7b, GCNF) was well validated by our CKE model, we observed mmu-let-7g, and mmu-let-7i are in the same cluster as mmu-let-7b (see Figure 5, top right). It was claimed that let-7b and 7g reduce tumor growth in mouse models of lung cancer [44]. We then checked that indeed these three miRNAs have very similar mature sequences, namely 

• mmu-let-7b: UGAGGUAGUAGGUUGUGUGGUU

• mmu-let-7g: UGAGGUAGUAGUUUGUACAGU

• mmu-let-7i: UGAGGUAGUAGUUUGUGCUGU

To evaluate the correlation between mature sequence and expression profile of our set of 266 miRNAs, we systematically explored all the 266×265/2=35,245 pairs (*mir*_*i*_,*mir*_*j*_) of distinct miRNAs in this set, *i*≠*j*,*i*,*j*=1,…,35245. For each such pair we then computed the Euclidean distance between the expression level profiles (expression distance in short) and the Needleman-Wunsch alignment score (NWA) between the mature sequences of each miRNA pair. We then divided the 35,245 miRNA pairs into two groups: GroupHigh includes the pairs with high alignment score (*NWA*>10), i.e. GroupHigh includes miRNA pairs with similar mature sequences, and GroupLow includes the pairs with low alignment score (*NWA*≤10). We then compared the distribution of expression distances for all miRNA pairs in GroupHigh with the distribution of these distances for miRNA pairs in GroupLow. As seen in Figure 5 (bottom left), the quantiles of these distances in GroupLow are consistently larger than the corresponding quantiles in GroupHigh. This is fully confirmed by Kolmogorov-Smirnov test which yielded the very significant p-value 7×10^-32^. The result still holds when we change the alignment score threshold *NWA*=10 used to define GroupHigh and GroupLow (see Figure 5, bottom right, where the NWA threshold is now *NWA*=15). We conclude that miRNAs having similar mature sequences tend, with high probability, to have similar expression levels. The above analysis and examples indicate that miRNAs belonging to the same cluster are good candidates to have similar mature sequence. Since the match between miRNA mature sequence and target sites is the main determinant for miRNA targets, miRNAs belonging to same cluster may hence also have similar regulatory roles.

### Robustness of parameter estimation

**Figure 6:**
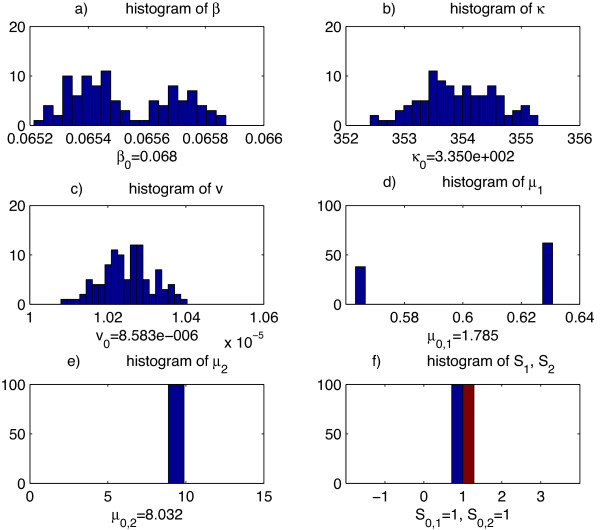
**Histogram of re-estimated parameters for a TD model. a)** the histogram of re-estimated parameter *β*, degradation rate of Sox2, the originally estimated *β* value of the model is below the graph. **b)** the histogram of re-estimated parameter *κ*, transcription rate of Sox2, the originally estimated *κ* value of the model is below the graph. **c)** the histogram of re-estimated parameter *v*, reaction rate of Sox2 and mmu-mir-21, the originally estimated *v* value of the model is below the graph. **d)** the histogram of re-estimated parameter *μ*_1_, reaction constant of Sox2 and Oct4, the originally estimated *μ*_1_ value of the model is below the graph. **e)** the histogram of re-estimated parameter *μ*_2_, reaction constant of Sox2 and Nanog, the originally estimated *μ*_2_ value of the model is below the graph. **f)** the histogram of re-estimated parameter *S*_1_,*S*_2_, number of binding sites of Oct4 and Nanog on Sox2 respectively, the originally estimated *S*_1_ and *S*_2_ value of the model is below the graph.

**Figure 7:**
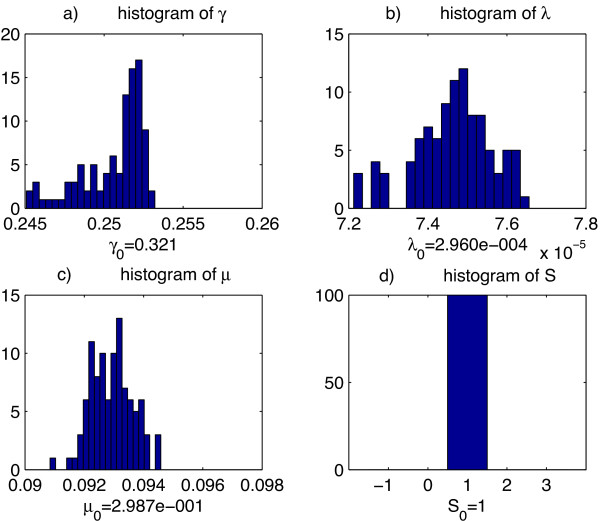
**Histogram of re-estimated parameters for a TI model. a)** the histogram of re-estimated parameter *γ*, degradation rate of GCNF, the originally estimated *γ* value of the model is below the graph. **b)** the histogram of re-estimated parameter *λ*, translation rate of GCNF, the originally estimated *λ* value of the model is below the graph. **c)** the histogram of re-estimated parameter *μ*, reaction constant of GCNF and mmu-let-7b, the originally estimated *μ* value of the model is below the graph. **d)** the histogram of re-estimated parameter *S*, number of binding sites of mmu-let-7b on GCNF respectively, the originally estimated *S* value of the model is below the graph.

Estimation algorithms for nonlinear models may yield parameter estimates that are dependent on the particular set of data or on initial estimates of parameters. Since our parameter estimation algorithm is independent from initial estimates of parameters, we now focus on on the measurements errors affecting microarray data and on their impact for parameter estimation. Considering that the noises of the microarray data are not negligible, we have analyzed the robustness of our parameters estimators when one perturbs randomly the observed expression levels of miRNAs. We have selected an arbitrary validated model *MD*, where the corresponding downstream factor is denoted as *D*, and has expression levels *d*(*t*). Denote miRNAs *M*_1_,…,*M*_*j*_ pertaining to model *MD* and call their expression levels *m*_1_(*t*),…,*m*_*j*_(*t*). Then we have perturbed the expression levels of the miRNAs by independent random noises having the recorded standard deviation *σ*_1_(*t*),…,*σ*_*j*_(*t*) and obtained simulated expression levels *sm*_1_(*t*),…,*sm*_*j*_(*t*). After injecting the perturbed expression levels *sm*_1_(*t*),…,*sm*_*j*_(*t*) into the model *MD* and get the predicted expression level *pd*(*t*) of *D*. With *pd*(*t*),*m*_1_(*t*),…,*m*_*j*_(*t*) and expression levels of other upstream factors, we then applied our parameter estimation algorithm to re-estimate the model parameters. This procedure was repeated 100 times. Then for each model parameter, we plotted the histograms of those 100 re-estimated parameter values and compared them with the parameter values estimated from unperturbed data of model *MD*. This analysis showed that our parameter estimation algorithm is quite robust. Here we present the histograms of perturbed estimates of model parameters for one TD motif (Figure 6) and one TI motif (Figure 7).

## Conclusion

We have separately modeled by chemical kinetics equations the 2 distinct modalities of the repressive actions of miRNAs on post-transcriptional processes of mRNA genes and the associated proteins. This was achieved by first defining the formal structure of two types of interaction architectures (Transcription Degradation motifs and Translation Inhibition motifs) linking miRNAs to subgroups of mRNA genes. The plausibility of each one of these potential TD motifs or TI-motifs was then evaluated by computerized parametric modeling, based on microarray data, of adequate formal chemical kinetics equations (CKEs).

We have sketched the formal derivation of 2 specific CKEs modeling by dynamic ODEs the interactions between concentrations of different species of molecules involved in each architecture. This led to a motif validation strategy based on the quantified quality of fit between our optimally parametrized models and the corresponding microarray data.

Our computerized parameter estimation is implemented by an innovative fast algorithm that does not require knowledge of range of molecular reaction rates. On a current standard laptop PC, our implementation of parameter estimation for a typical 9-parameters CKE model requires about 5 minutes of computing time.

Our parameter estimation algorithm also provides relatively high-quality optimization for the fit between model and microarray data, by integrating both global and local cost minimization techniques, in contexts where plausible ranges of values for most of the unknown parameters are not available in the literature. By perturbing the expression levels of miRNAs and re-estimating the parameters, we showed that our parameter algorithm has a satisfactory level of robustness. We believe that our parameter estimation technique with associated evaluation of quality of fit would be quite applicable as a generic algorithm to similar problems in chemical kinetics modeling of molecular interactions.

Modeling very large microarray data is computationally quite expensive. We have hence sketched clustering methods to condense large microarray data. This approach has of course been attempted before our work, but the main point is that we have carefully studied the mathematical compatibility of our CKE models with condensation of the profiles data. Since we have proved that the abstract form of our CKE models is invariant by arbitrary multiple affine transformations of profiles data, we have made sure to constrain the distance of two expression levels profiles to be invariant by these types of affine transformations.

We have implemented a Minimal Net Clustering algorithm based on this distance, which allows us to control the radius of the clusters. The number of CKEs to parameterize can be strongly reduced after condensation of the large data sets, and the affine invariance of our CKEs show that the condensed genes network can then still be modeled by similar CKEs.

By applying our TI modeling to multiple proteins such as GCNF, Oct4, Nanog, Sox2, we showed that 3 miRNA-target pairs experimentally validated can be also validated by the TI model.

## Competing interests

The authors declare that they have no competing interests.

## Authors’ contributions

ZL developed the chemical kinetics equations and the parameter estimation algorithm; RA proposed the transformation invariance of chemical kinetics equations and data condensation method; YZ did the computation work. ZL and RA wrote the paper. All authors read and approved the final manuscript.

## Supplementary Material

Additional file 1 Codes-minimal net clustering. Click here for file

Additional file 2 Codes-parameter algorithm. Click here for file
